# Evaluating case studies of community-oriented integrated care

**DOI:** 10.1080/17571472.2018.1477455

**Published:** 2018-05-31

**Authors:** Paul Thomas, Amrit Sachar, Andrew Papanikitas, Alison While, Chris Brophy, Chris Manning, Cliff Mills, Baljeet Ruprah-Shah, Catherine Millington-Sanders, David Morris, Deirdre Kelley Patterson, Diana Hill, Emma McKenzie-Edwards, Fiona Wright, Francesco Carelli, Freddy Shaw, Isabelle Vedel, John Spicer, Liz Wewiora, Malik Gul, Michelle Kirkbride BA, Mike Sadlowski, Mylaine Breton, Ricky Banarsee, Sunjai Gupta, Tony Burch, Tulloch Kempe, Victoria Tzortziou Brown, John Sanfey

**Affiliations:** a London Journal of Primary Care, RCGP, London, UK; b Clinical and Implementation Lead for Mental Health in North West London Diabetes Transformation Programme, North West London Collaboration of CCGS, London, UK; c Ethics and Philosophy, London Journal of Primary Care, NIHR Academic Clinical Lecturer in General Practice, Oxford University, Oxford, UK; d Community Nursing, King’s College London, London, UK; e Resolving Together Limited, RCGP, London, UK; f UPstream Healthcare Action for NHS Workforce Wellbeing, RCGP, London, UK; g Principal Associate Mutuo, Consultant with Anthony Collins Solicitors, London, UK; h Accomplish Consultancy, Programme Lead for Transformation, Kensington and Chelsea Social Council, RCGP, London, UK; i Kingston CCG End of Life Care Clinical Lead and Macmillan GP, RCGP, London, UK; j Mental Health, Inclusion and Community, Centre for Citizenship and Community School of Social Work, Care and Community, University of Central Lancashire, Preston, UK; k Centre for the Study of Policy and Practice in Health and Social Care, University of West London, London, UK; l Essential Parent, London, UK; m London Journal of Primary Care, University of Oxford and Salaried GP, Oxford, UK; n Public Health, London Borough of Barking and Dagenham and Greater London Authority, London, UK; o London Journal of Primary Care, Professor Family Medicine, EURACT Council Executive Board, University of Milan, Milan, Italy; p Medical Educator, RCGP, London, UK; q Department of Family Medicine, McGill University, Montreal, Canada; r Head of Primary Care Education and Development, Health Education England [South London Team], London, UK; s Open Eye Gallery, Liverpool, UK; t Wandsworth Community Empowerment Network, London, UK; u NorWest Co-op Community Health Centre, Winnipeg, Canada; v BKin, MSc Applied Ergonomics, Winnipeg, Canada; w Department of Community Health, Universite de Sherbrooke, Sherbrooke, Canada; x WeLReN CiC, London, UK; y South London and Maudsley NHS Trust, London, UK; z Health Education England North London, London, UK; aa Service Development, Staywell, Kingston Coordinated Care Programme – Design Team and Delivery Group member, London, UK; ab Lead On Integrated Care and Research NHS Tower Hamlets CCG and RCGP Joint Hon Sec, RCGP, London, UK; ac Hammersmith and Fulham GP Federation, London, UK

**Keywords:** Community-oriented integrated care, new care models, primary care home, community-based co-ordinating hubs

## Abstract

This paper summarises a ten-year conversation within London Journal of Primary Care about the nature of community-oriented integrated care (COIC) and how to develop and evaluate it. COIC means integration of efforts for combined disease-treatment and health-enhancement at local, community level. COIC is similar to the World Health Organisation concept of a Community-Based Coordinating Hub – both require a local geographic area where different organisations align their activities for whole system integration and develop local communities for health. COIC is a necessary part of an integrated system for health and care because it enables multiple insights into ‘wicked problems’, and multiple services to integrate their activities for people with complex conditions, at the same time helping everyone to collaborate for the health of the local population. The conversation concludes seven aspects of COIC that warrant further attention.

## Related LJPC Papers

• Elisabeth Paice & Samia Hasan (2013) Educating for integrated care, London Journal of Primary Care, 5:1, 52–55, DOI: 10.1080/17571472.2013.11493374
• Paul Thomas, Tony Burch, Ewan Ferlie, Rachel Jenkins, Fiona Wright, Amrit Sachar & Baljeet Ruprah-Shah (2015) Community-oriented integrated care and health promotion – views from the street, London Journal of Primary Care, 7:5, 83–88, DOI: 10.1080/17571472.2015.1082347
• Thomas P (2017). Collaborating sites for community-oriented integrated care and health promotion, London Journal of Primary Care, 9:1, 2–6, DOI: 10.1080/17571472.2016.1271491.
• Victoria Tzortziou Brown, Irem Patel, Nicola Thomas, James Tomlinson, Rachel Roberts, Hugh Rayner, Neil Ashman & Sally Hull (2017) New ways of working; delivering better care for people with long-term conditions, London Journal of Primary Care, 9:5, 60–68, DOI: 10.1080/17571472.2017.1361619
• John Sanfey (2017) The use of case studies to drive bottom-up leadership in community-oriented integrated care and health promotion (COIC), London Journal of Primary Care, 9:1, 7–9, DOI: 10.1080/17571472.2016.1271497

## Why this matters to us

London Journal of Primary Care (LJPC) is a network of people who want to develop community-oriented integrated care. This includes building, as a force for whole-society health, local health communities. Such communities see health as an asset to build from, as well as treating diseases where possible. Over ten years we have co-produced papers that reveal different insights into COIC, and have had tangible impact on policy. This stage of LJPC has come to a natural end. We want to describe what we have learned, and how others can continue this kind of work in different contexts.

## Key message – What the reader might learn from the paper

How to develop case studies of community-oriented integrated care and health promotion.

## Governance

LJPC Board facilitated discussions and provided overview of the writing of this paper

On 13 March 2017 nineteen participants at a London Journal of Primary Care (LJPC) meeting in London (UK) discussed aspects of community-oriented integrated care (COIC) that need to be better understood. They continued a conversation of many years. In the very first year of LJPC (2008) nearly half of the 52 papers explored the modern-day implications of the 1978 Alma Ata agreement. This identified community-oriented integration of care and health improvement to be a key component. In 2012 and 2013, with colleagues from America and Canada, LJPC stimulated debates about ‘Local Health Communities for Integrated Care’ at the North American Primary Care Research Group (NAPCRG) [1]. In 2015 and 2017, with RCGP London, LJPC stimulated debates about ‘Community-oriented integrated care and health promotion’ at the (London) City Health Conferences [2]. In 2017 LJPC started to develop a network of collaborating sites to better understand how to achieve community-oriented integrated care and health promotion in different contexts [3].

Participants in the conversation have included colleagues from Canada and America as well as the UK. Many are primary care practitioners. Others have specialist expertise in mutual organisations, public health, research, nurse leadership, end of life care, schools, the arts, self-care, patient self-help groups and community development initiatives.

Arriving at a shared understanding of COIC took time. Perhaps it was the language of the time that led to its emergence. The popular rhetoric was ‘integrated care’; to which the response of the LJPC community was: *yes, and integration must be oriented towards local communities rather than towards hospitals*.

COIC is a concept rather than a specific model. As well as vertical integration between specialists and generalists through ‘care pathways’, it advocates horizontal integration between primary care and public health, as emphasised in *community*-*oriented primary care* [4]*,* the *New Public Health* [5] and community-oriented approaches to general practice [6]*.*

COIC values health as an asset to build healthy societies, as well as appreciating the need to treat illnesses [7]. COIC combines structural alignment, as emphasised in the World Health Organisation (WHO) concept of a geographic, *community*-*based coordinating hub* [8] with the *learning organisation* principle of cycles of collaborative learning and coordinated change to build healthy communities [9–11]. COIC also involves collaboration between groups of people with different interests (e.g. patients, citizens, healthcare professionals), and across organisational boundaries, helping to develop shared purpose for health within communities. It therefore benefits from theories, models and structures that support co-operative working [12].

Considering health to be an asset from which to build healthy societies, while also treating illnesses is a *wicked problem.* This is a problem that is *difficult or impossible to solve because of incomplete, contradictory, and changing requirements that are often difficult to recognise*. Wicked problems cannot be solved through one agent; they require inter-organisational and inter-personal collaboration to address more than one thing at the same time, and they require co-adaptation for things to fit well together. This means that COIC is closely linked to the idea of a *local health community,* namely a geographic area where different disciplines and all citizens collaborate for the sake of the health of the population as a whole. This requires a shared vision that is broad enough to be meaningful to all (i.e. whole society healthy), a system that shows how different people can contribute at different times, and a broad sense of ownership that is generated when people engage in cycles of collaborative learning and coordinated change.

## 1. International conversations about community-oriented integrated care

Ever since the 1978 WHO conference at Alma Ata, countries throughout the world have recognised that efforts for care and health improvement need to be integrated at local, community level to produce a healthy society. It came to be called ‘Strong’ or ‘Comprehensive’ Primary Health Care [13]. This was reaffirmed in 2008 when the WHO Director General Margaret Chan again advocated *community*-*based coordinating hubs* – *reorganisation of health services as primary care … coordinator of a comprehensive response at all levels……that secures healthier communities, by integrating public health actions with primary care and by pursuing healthy public policies across sectors* [8]*.*

Throughout the world, in one way or another, since 1978 the concept of COIC has been advanced. There are now 1400 Healthy City Programmes which are working towards the ideal of whole society health. The latest phase of the Healthy City movement, from 2018, will emphasise the six ‘P’s – People, Place, Participation, Prosperity, Peace and Planet. These will feature in the Alma Ata 40th Anniversary Conference in October 2018 (in newly-named Almaty) that will also call for the support of 20,000 elected city mayors and political leaders who advocate the six ‘P’s by the year 2020.

In the UK, with the Five Year Forward View, COIC has finally come of age. It is an intrinsic part of the Accountable Care concept, more recently termed *Integrated Care Systems,* currently being implemented throughout the UK. It reflects explorations by all political parties of a ‘new localism’ where naive ‘top down’ control and ‘bottom up control’ are both replaced by a more equal and dialogic relationship between local and central functions [14].

The current commissioning paradigm is likely to be replaced with one based on capitation budgets that will be shared between provider organisations in Primary Care, Acute and Community Trusts, and Social Care. This new paradigm focuses on whole population outcomes. COIC is an essential and hitherto undervalued component in the integration of health and social care services, intended to be delivered at local, community level through *community networks*. The word ‘local’ is important here, because the range of things that affect health are part of the fabric of lives being lived. Further away, for example in a hospital, the focus tends to be on the disease rather than the person. ‘Community’ is important because trusted relationships between multiple agencies can have effects more than the sum of the parts. A geographic locality is important to provide a shared developmental space where different organisations can creatively interact.

Secondary Care will probably hold the lion’s share of capitated budgets within Integrated Care Systems. However, these new contracts will be judged by population health outcomes, and will have longer time-spans with less emphasis on in-year balancing of budget. This should motivate the development of COIC strategies that promote health and prevent disease, as well as treat illness. Integrated (Accountable) Care could boost existing COIC strategies already being developed through the models of Community Development Agencies [15], Primary Care Homes [16], New Care Models and Vanguard Sites [18]. In the West London Integrated Care Programme, movement towards COIC has been evidenced by the development of Health Networks (when collaborating for medical matters) and Local Health Communities (when collaborating for broader health concerns) [19–21].

In the UK, the role of Clinical Commissioning Groups (CCGs) in Integrated Care Systems will be to determine the population health outcomes against which the new contracts will be judged. In addition to the short term, focused outcomes of treating disease, evaluation of COIC will need to consider longer-term and broader impacts including the wider determinants of illness AND the wider determinants of health, local communities for health, sustained collaborative efforts, whole system impact, unexpected outcomes, and improved resilience of the individuals and communities involved.

## 2. Case studies of community-oriented integrated care

Community-oriented integrated care is a general concept rather than a specific model. Making it work well means applying it wisely to the specific context, preferably in a way that allows co-evolution rather than starting an entirely new system. A *case study* describes the history of a particular set of actions, how and why they were chosen, and their consequences. It is a good way to tease out the interplay of local context and generalizable principles when implementing policy [22].

A network of collaborating sites could nurture case studies to help to understand COIC. These are places that are prepared to contribute to a shared exploration of the meaning of COIC in different contexts. Sometimes a case study will be the collaborating site; sometimes a collaborating site will relate to a number of case studies. Key to maximising the learning from these networks will be a publishing platform to locate papers about the cases and stimulate discussions between sites about the lessons arising from them.

To build a rich picture of case studies, the publishing platform should link papers relating to each site, including papers published in other places. To track the evolution of ideas across larger areas it should enable inter-site conversations. This sharing of ideas and experiences about a theme could include academic ‘Reviewed’ papers that use a recognisable evaluation methodology and ‘Landscape’ papers that provide anecdotes and stories. One method that LJPC has been exploring includes week-long (Google Plus) discussions of the policy implications of a set of published papers, coupled with hour-long ‘Twitter Storms’ – summaries then curated by a multidisciplinary team. The publishing platform should also help researchers to develop an appropriate research methodology [22] and support authors to write well.

Within a case study, a core leadership team should ‘hold’ the overall story and use methods that enable deep and ongoing discussion within the site itself, using all available data including lived experience. As well as evaluating structures and outcomes, case studies should evaluate the co-creative processes of COIC that build communities and integrate different processes. They should track how this activity informs policy for whole society health and reveals new research needs. Evaluation of such broad and inter-linked activities cannot be done with simple measures. It requires multi-method, multi-sector, whole systems research. Guba and Lincoln propose that ‘4th Generation Evaluation’ [23] has the power to do this – it is a form of participatory action research that generates insights from the three inquiry paradigms of positivism, critical theory and constructivism (quantitative and qualitative insights) and subjects all to broad stakeholder critique at a series of stages to shape the next stages of the emerging story.

Case studies would include a foundation paper that describes their story, including their social and political context as well as service configurations. This describes their unique context. They can later refer back to this paper as the story unfolds. For example, a foundation paper could describe:

• The goal of the programme, and how the goal came to be established
• Partner organisation roles and boundaries, including community and voluntary sectors
• Disciplinary & organisational participation and their leadership institutions
• Funding sources and flows
• Morbidity and demographics
• Evaluation and key metrics
• Theories and models of change
• Implementation strategies, barriers and facilitators
• Training and skills development
• Wider determinants of health and illness, including employment and employability
• Health education initiatives
• What they can contribute to local and national policy

Later papers from a case study would develop the story and share key learning to contribute to the debate on the development of COIC. They could also show change in sets of routinely-gathered data alongside other sources of information, through sequential cycles of development.

## 3. Elements of COIC that need better understanding

The following aspects of COIC are insufficiently understood, and need further exploration:

### 3.1. Different models of integrated working

Case studies of COIC need to clarify their theories of change and models of action, not only adapting the principles to their changing local contexts but also ensuring they are understood by other disciplines within their community of interest.

They also need to clarify whether their aims are to Integrate: (a) medical practice, (b) multidisciplinary teams, or (c) networks, communities and systems. Mead’s 2006 study of primary care innovation in 31 countries provided an important analysis of primary care systems across the world that still has important lessons for today. It revealed six ideal types of primary care organisation, namely outreach franchise; reformed polyclinic; extended general practice; district health system; managed care enterprise; community development agency. Of these, the *community development agency* most obviously develops communities for health, beyond medical care. Meads examined case studies in Peru, Costa Rica, Venezuela and Bolivia, in which they maintained: *health is a citizen, not a profession issue* (p. 100) [15].

Further analysis of Mead’s work revealed that his six ideal types naturally cluster into three models of different kinds of integration that case studies might recognise. Evaluation and lessons learned could consider the extent to which case study sites relate to these models:

Model 1: Outreach franchise and polyclinic – integrating through medical practice.
Model 2: Extended general practices and district health systems – integrating through multidisciplinary teams.
Model 3: Managed care and community development agencies – integrating through networks, communities and systems [24].

### 3.2. Policy for whole system collaboration

COIC requires team-working across organisational and disciplinary boundaries that helps a wider system to work as an integrated organic whole. In the UK, evaluation could explore the extent to which transition from a commissioning model of health care to the new shared capitation budget (a) facilitates such multiple-way team-working, (b) enables people to feel able to help themselves and others, and (c) builds communities and networks for health.

Evaluation could focus on:

• Ways in which policy and interventions stimulate individuals and communities to see health as an asset to build healthy economies and healthy societies.
• Ways in which policy stimulates shared-care, self-care and local communities in which citizens and practitioners routinely care about the health of others, beyond their own self-interest.
• Ways in which organisations apply principles of organisational learning to whole systems, including cycles of inter-organisational learning and change and facilitation of collaborative improvements in different parts of the system.
• Knowledge, skills and attitudes that help citizens to participate effectively in community development initiatives, and ways to re-learn these things at different stages of life.
• Mechanisms for co-creative interaction, including strategic partnerships for sustainability, adaptation to new challenges and ways to ‘invest to save’.
• Ways in which theories and models from the co-operative movement help people to actively participate in health and well-being, and build communities and networks for health and well-being.
• Organisational and structural arrangements which enable people to collaborate across organisational and disciplinary boundaries.
• Ways in which evidence-based primary prevention is commissioned and put it in the context of community-oriented integrated care [25].

### 3.3. Outcomes

In 2015 the Royal Society of Arts and its partners at the University of Central Lancashire (UCLan) and the London School of Economics (LSE) published the findings of a three year study on *Connected Communities* [26]*.* They identified four outcomes or ‘dividends’ from interventions designed to reveal and build community networks, each with implications for health:

(a) *A wellbeing dividend*. Social connectedness correlates strongly with wellbeing
(b) *A citizenship dividend*. Enhancing the citizenship opportunities of community members can improve health
(c) *A capacity dividend*. The impact of improved health spreads through networks to build the capacity of others for improved health and wellbeing
(d) *An economic dividend*. Social relationships improve employability, civic participation as well as health, enabling potential for cost benefit in health and welfare.

Now being further evidenced by way of a parallel series of trans-disciplinary action research projects in the work of Prof. David Morris and UCLan’s Centre for Citizenship and Community, these dimensions of community capital represent categories that may be adapted to help identify data for case study sites to routinely generate, in order to evaluate the overall effect of complex interventions:

• *Wellbeing:* Use of population data to develop preventive strategies, predict frailty and use social interventions to enhance welbeing. New models of care planning and more proactive approaches to chronic disease management. Change in tracer markers (e.g. blood sugar control). Markers of health including action competence and social-connectedness that reveal the degree to which people have positive life stories and networks of relationships.
• *Citizenship:* Self-care. Participation in improvement projects. Co-produced Social Prescribing models. Use of services by patients who have and don’t have a Care Plan.
• *Capacity:* Patient and Staff satisfaction and competence. Networks of multidisciplinary leadership teams and stakeholder feedback of their effectiveness.
• *Economic:* Unscheduled consultations and admissions of patients with and without a Care Plan. Risk to patients, staff and the system as a whole need to be continually assessed and anticipated, to provide optimal safety.

Many public service organisations, including general practices and hospitals, routinely code clinical episodes. Data can be amalgamated from these databases to measure changes in many of the above outcomes, by practice, locality and larger areas like boroughs and cities.

### 3.4. Interaction between organisations within a community of interest

Individual organisations such as general practices, have a set of tasks to undertake, and an organisational purpose to fulfil. These tasks can be done in ways that enhance or diminish relationships with other organisations and disciplines. Organisations that have shared purpose within a community of interest can work collaboratively to improve a range of issues. Case studies can describe the mechanisms they use to collaborate and to ensure that those tasks enhance relationships with other disciplines and enable individuals and teams to span organisational boundaries. For example, (a) Seasons of activity coupled with an annual calendar of events help different organisations to align their ways of working, (b) Formal agreements support a series of complex collaborations, (c) Live Manuals help to remind everyone of the things they have agreed to do, (d) Local discussions about data assist real-time evaluation and co-adaptation [27].

Evaluation could focus on:

• *Local health communities.* Models and interventions that support the development of community-based coordinating hubs as local communities for health, including measures of social cohesion.
• *Care pathways into and out of community hubs.* The cost and quality of care pathways could be monitored, to include numbers, speed of access, satisfaction and diseases treated.
• *Adequacy of professional roles.* The rationale for new professional roles could be described and their effect piloted.
• *Mechanisms for collaboration.* Organisational and structural arrangements for collaboration and co-operation across organisational boundaries can be explored and evaluated.

### 3.5. Infrastructure of facilitation and communication

To make COIC happen at scale and be sustained over time, case studies need an infrastructure of communication and facilitation that supports projects, generates data and develops leaders to advance collaboration. These need to support both hospital and community practitioners and managers, and be explained in languages that each can relate to. For example, medical specialists are likely to appreciate the value of collaboration to prevent diseases, but less easily see the value of social cohesion as a health asset in its own right. Evaluation could focus on:

• *Applied research units* that support all aspects of collaborative improvement, using fourth generation evaluation to facilitate multi-perspective evaluation [23].
• *Mechanisms* in different parts of society, beyond healthcare, to support boundary-spanning learning and change for shared care, health promotion and community development.
• *Large group formats.* Large group events such as Open Space [28] Future Search [29], Appreciative Inquiry [30] and Real Time Strategic Change [31] help large numbers of people from different disciplines to creatively interact.

### 3.6. Strategies to develop and sustain leadership

Leadership for COIC combines sense-making [32] with ‘heroic’ individual actions. Networks of multi-disciplinary leadership teams, including specialists and generalists, can help various constituencies to make sense of the complexity that might otherwise overwhelm them. They can help people to reach out rather than retract. Evaluation could focus on:

• *Networks of multidisciplinary leadership teams*. Leadership teams need to be recruited and supported to learn how to engage people and organisations in coordinated cycles of inter-organisational, inter-disciplinary learning and change and link these to established functions such as professional bodies, policy-making organisations and funding bodies.
• *Community development*. Leaders need to understand and meaningfully apply community development principles. They need to understand that cooperative working often happens when people cannot get ‘market share’ and can cease when a new way of working is established so ongoing renewal is an essential aspect of sustainability.
• *Learning*. Support for leaders and leadership teams is needed to help them to devise, adapt and lead models that engage communities and help them to develop themselves and to communicate the value of their work to others. Initiatives can be led from various places, including schools and voluntary agencies (e.g. Hans Kai patient self-help groups).
• *Continual quality improvements*. Support for leadership teams could include ongoing learning about how to lead continual system-wide improvements, and succession planning. Such support needs to be targeted at a range of disciplines and their academic homes, including nursing and allied health professions, social care and voluntary groups.
• *Education sector* needs to include skills to lead and evaluate community-oriented integrated care on various curricula, including those of health professionals.

### 3.7. Ways for citizens to learn the skills to take part

‘Horizontal’ aspects of COIC require trusted relationships in multiple directions. This can conflict with the present emphasis on individualism and compartmentalisation. Citizens and healthcare practitioners alike need to learn and re-learn how to value diversity and build resilient communities. This is needed at all stages of life, including formative and ongoing learning of health workers, new parents, primary and secondary schools, working life and the Third Age. Society needs to develop citizens who are ready, willing and able to contribute to a healthy society. Evaluation can focus on the knowledge, skills and attitudes needed to do this, including people’s ability to:

• Be alive and balanced in the moment, able to adventure in unfamiliar places, be a team player, and a resilient, life-long, life-wide learner.
• Be able to conceptualise whole systems and coherent stories, see connections between parts and wholes and make timely contributions to enhance both.
• Be able to develop equal relationships and diverse communities.
• Participate formally as actors within an organisation (e.g. as members of a co-operative), and thus enable citizens as a constituency to have a voice alongside healthcare practitioners.

## 4. Approaches to evaluation

Throughout the world, ways to evaluate community-oriented integrated care are being considered both as a theoretical approach to systems research [33] and as practical evaluation of contemporary models such as the UK New Care Models [34] and Primary Care Homes [17].

As well as outcomes, processes of engagement that build communities of interest need to be evaluated. For example, the number of members and partners involved and what they practically do; conversations held and concluded, co-produced papers published then used to stimulate more debate and more co-production. The responsibilities of members and collaborating organisations need to be clear and the degree to which they fulfil their roles audited. One reason for this is that the ‘magic’ within COIC goes beyond mechanical efficiency (although that is important). The magic lies in the degree to which the process motivates participants to be creative and self-actualised, see their parts in bigger wholes, feel that they belong and are able to build trusted relationships.

## 5. Conclusion

We need to transform health-care services, which treat illnesses one at a time through individual actions, into integrated health and care systems where community-oriented integrated care (COIC) facilitates a whole society quest for whole society health. Everyone needs to be skilled at shared-care and self-care. Everyone needs to be a team-player, system-thinker and community-developer, and everyone needs to support the development of these skills in everyone else.

LJPC has facilitated rich multidisciplinary debates over ten years about how to achieve COIC. We believe that the time is now right to set up a network of collaborating sites to pilot and evaluate case studies of COIC at scale. Case studies need to evaluate care pathways, collaborative health improvements and stakeholder engagement. This paper describes seven areas to pay attention to.

A network of collaborating sites could be thought of as interlinked *communities of interest,* united in a shared purpose to shift culture away from naïve individualism towards a healthy balance between valuing individual selves and appreciating others, and between responsibilities and rights [35]. People should expect ‘Both-And’ solutions to complex issues, and be appropriately wary of simple linear and controlling ‘Either-Or’ solutions outside of short-term, transactional tasks.

From primary schools to university post-graduate programmes, students need to learn and re-learn these skills and be able to evaluate complex interventions using multi-paradigm inquiry that builds communities and reveals different aspects of whole moving pictures.

We should be alerted to the difficulty of this transformation by the fact that 2018 sees the 40th anniversary of the international Alma Ata declaration that agreed that these things are needed in healthy societies and in a healthy world. Yet, they have happened in only patchy ways and the theories, concepts and models for success remain contested.

We hope that the 70th anniversary of the NHS and the 40th anniversary of the WHO Alma Ata Declaration will spur many to take forward these ideas.

## Endorsements

The following endorse the ideas presented in this paper.

Dennis Ougrin. Consultant Child and Adolescent Psychiatrist. Clinical Senior Lecturer. Course Leader, MSc in Child and Adolescent Mental Health. Child and Adolescent Psychiatry. Institute of Psychiatry. PO85 De Crespigny Park. London SE5 8AF. 44(0)2078480957. E-mail: dennis.ougrin@kcl.ac.uk

John R Ashton. Professor. Former President of the United Kingdom Faculty of Public Health and Chair of the UK Public Health Association. E-mail: johnrashton@blueyonder.co.uk

John M. (Jack) Westfall, MD, MPH. Chair of Family Medicine. Santa Clara Valley Medical Center Health and Hospital System. San Jose, CA. E-mail: Jack.westfall@ucdenver.edu

Linda Lang. Professor. Senior Advisor. Baker Dearing Educational Trust. 4 Millbank, London SW1P 3JA. E-mail: llang@utcolleges.org

Mark Spencer. Medical Director North West London Collaboration of CCGs. Expert Principal, CarnallFarrar. E-mail: Mark.spencer@nhs.net

Peter J Whitehouse. Professor. Case Western Reserve University, Cleveland USA and University of Toronto. E-mail: peter.whitehouse@case.edu

Ricky Banarsee. Professor. Director West London Research Network (WeLReN CiC). E-mail: r.banarsee@imperial.ac.uk

Sarah-Jane Lang. General Practice Speciality Trainee, St George’s University of London. E-mail: Sarah-jane.lang@nhs.net

Siobhan Clarke. Managing Director of Your Healthcare CIC, London. E-mail: siobhan.clarke@yourhealthcare.org

Steve Thomas. Clinical Director. Mental Health, Learning Disabilities & Dementia Portfolio. NHS Sheffield Clinical Commissioning Group. E-mail: steve.thomas5@nhs.net 

Steve Iliffe. Emeritus Professor of Primary Care for Older People, University College London. E-mail: s.iliffe@ucl.ac.uk
